# Autoimmune encephalitis with coexistent LGI1 and GABA_B_R1 antibodies: case report

**DOI:** 10.1186/s12883-021-02460-w

**Published:** 2021-11-27

**Authors:** Yi Xie, Jia Wen, Zhihua Zhao, Hongbo Liu, Nanchang Xie

**Affiliations:** 1 Department of Neurology, The First Affiliated Hospital of Zhengzhou University, Zhengzhou, 450052 China; 2 Department of Orthopedics, The First Affiliated Hospital of Zhengzhou University, Zhengzhou, 450052 China

**Keywords:** Multiple auto-antibody, Autoimmune encephalitis, Anti-LGI1, Anti- GABA_B_R1

## Abstract

**Background:**

Autoimmune encephalitis (AE) with multiple auto-antibodies is of great clinical significance because its complex clinical manifestations and atypical imaging increase the difficulty of diagnosis, differential diagnosis and treatment, which may aggravate the disease, increase the recurrence rate and mortality. The coexistence of anti-Leucinie-rich Glioma Inactivated 1 (LGI1) and anti-γ-aminobutyric acid-beta-receptor 1 (GABA_B_R1) has not been published before.

**Case presentation:**

We herein present the case of a 60-year-old man with slow response, behavioral changes, psychosis and sleep disorders. Laboratory test included serum hyponatremia, positive serum LGI1 and GABA_B_R1 antibodies using transfected cell-based assays. Electroencephalogram exhibited moderate diffusion abnormality. The patient responded well to steroid impulse treatment and sodium supplement therapy, and did not recur during the follow-up.

**Conclusions:**

Here we report the first AE characterized by positive LGI1 and GABA_B_R1 antibodies, as well as summarizing AE with multiple auto-antibodies reported so far, hopefully to provide experience for clinical practice.

## Background

There are basically two kinds of auto-antibodies related to autoimmune encephalitis (AE). One is against neuron surface receptor, among which anti- N-methyl-D-aspartic acid receptor (anti-NMDAR) is the most common, others also including anti-γ-aminobutyric acid-beta-receptor (anti-GABA_B_R), anti-contactin associated protein-like 2 (anti-CASPR2), anti-α-amino-3-hydroxy-5-methyl-4-isoxazolepropionic acid receptor (anti-AMPAR), anti-Leucinie-rich Glioma Inactivated 1 (anti-LGI1), etc. The other kind is against neuronal intracellular antigen, mainly referring to classic paraneoplastic neuropathy antibody, such as anti-Hu, etc. [1, 2]. The majority of AE patients have only one of the above auto-antibodies, and very few have multiple auto-antibodies.

Different types of auto-antibodies correspond to specific neurological syndrome, which has strong specificity or directivity for etiological diagnosis. Both LGI1 and GABA_B_R1 are autoantigens of treatment-response limbic encephalitis, whose clinical manifestations include the rapid development of mood changes, depression, anxiety and dramatic loss of short-term memory [3]. LGI1-AE is characterized by confusion, cognitive impairment, sleep disorder, refractory hyponatremia, fascio-brachial dystonic seizures and high signal in medial temporal lobe and hippocampus [4]. The symptoms of GABA_B_R1-AE include cognitive dysfunction, seizures and abnormal behavior [5]. The simultaneous occurrence of both antibodies has not been reported before. Herein we report a case of a 60-year-old man with positive anti-LGI1 and anti-GABA_B_R who improved greatly after steroid therapy. We aim to remind physicians of this rare AE case with multiple auto-antibodies in potential clinical context.

## Case presentation

A 60-year-old Chinese male has developed slow response, abnormal behavior and sleep disorder for 1 month. At first, after admitted to the hospital in his hometown and given only sodium supplement and support treatment, his symptoms disappeared but quickly reoccurred. After that, his symptoms became more and more serious and he gradually developed seizures and irritability. He demonstrated confusion, memory loss, insomnia and abnormal behavior when transferred to our hospital in April 2020.

He had no particular previous medical history except for typhia 40 years ago and recovered with no sequel. Neurological exam revealed poor mental state, slow response and damaged memory, attention, calculation and orientation. Cranial nerves, cerebellar function, motor system, sensory system, deep tendon reflexes and pathological reflexes remained normal.

**Fig. 1:**
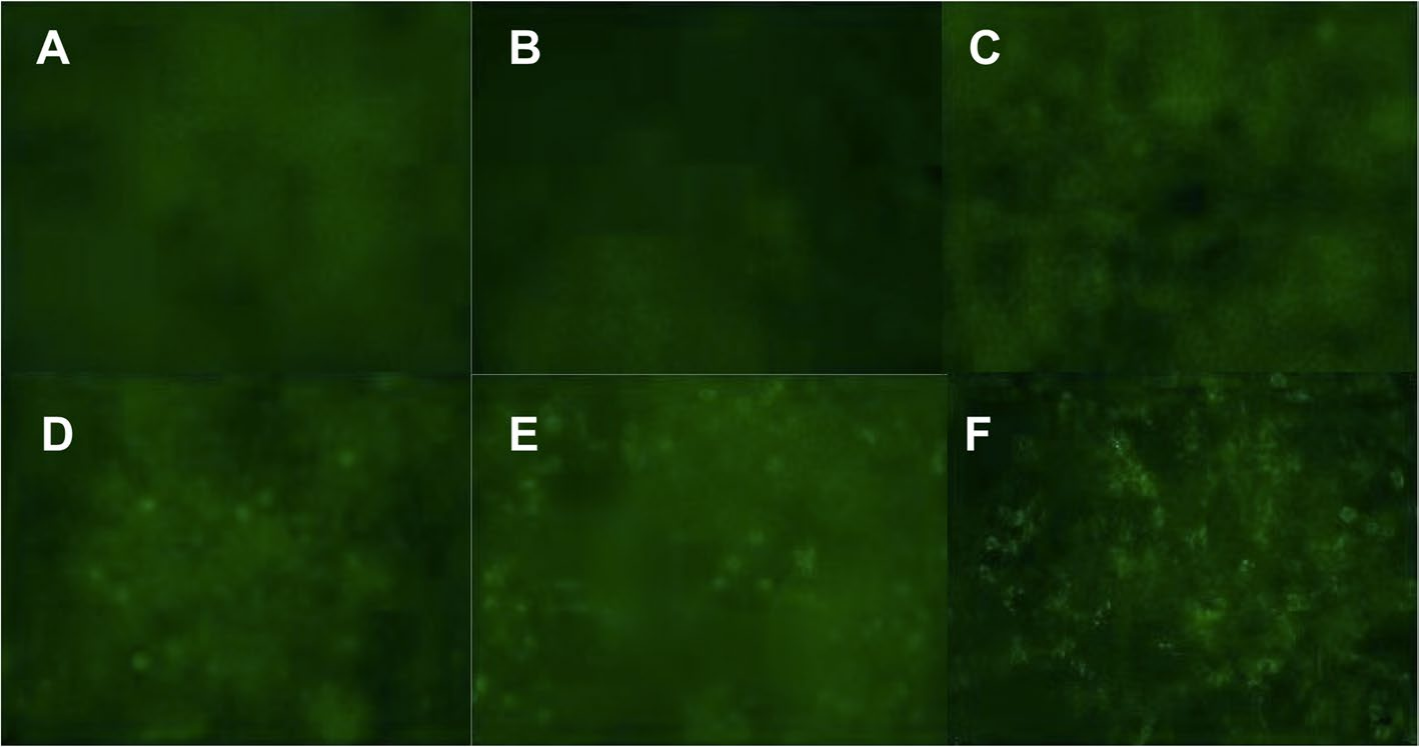
AE-related auto-antibodies in serum measured by cell-based assays. **A** NMDA; **B** AMPA1; **C** AMPA2; **D** CASPR2; **E** LGI1; **F** GABA R1. Anti-LGI1 and Anti-GABA_B_R1 were positive

**Fig. 2:**
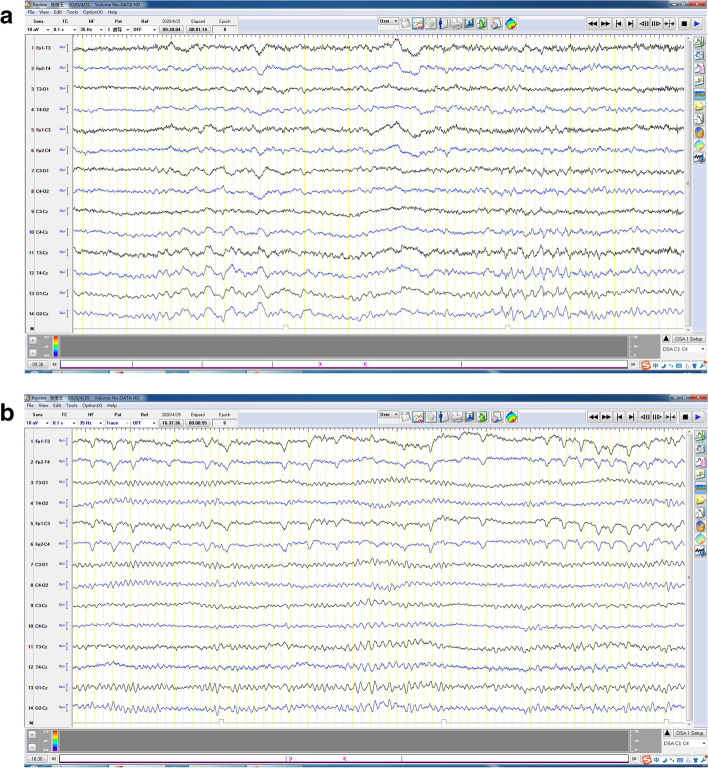
**A** EEG of the patient before treatment (2020-04-21): moderate diffusion abnormality, Wide range of slow waves occur in medium-high waves. **B** EEG of the patient after treatment (2020-04-29): normal

Serum sodium was 119 mmol/L (reference range: 135 ~ 153 mmol/mL) and chlorine was 81 mmol/L (reference range: 90 ~ 110 mmol/L) at first admission. Serum procalcitonin was 0.048 ng/mL (reference range: < 0.046 ng/mL), C reaction protein was 8.05 mg/L (reference range: < 5 mg/L). Cerebrospinal fluid (CSF) electrophoresis IgG index was 0.71 (reference range: 0.3 ~ 0.7). Intracranial pressure was 150mmH_2_O. CSF routine biochemistry for protein content and glucose were normal and infectious test for virus, including herpes simplex virus, tuberculosis, fungal and Cryptococcus were negative. CSF cytology and cytometry were negative for malignant cells. Serum AE antibody spectrum demonstrated positive anti-LGI1 IgG and anti-GABA_B_R1 IgG using cell-based assays, while other AE-related auto-antibody, such as anti-NMDAR, anti-AMPAR1, anti-AMPAR2, anti-CASPR2 were negative (Fig. 1). Mini mental state examination score was 15. Electroencephalogram (EEG) indicated moderate diffusion abnormality (Fig. 2A and B). Brain enhanced MRI scan was normal. Tests for screening malignancy, including tumor markers and an ultrasound of the liver, gallbladder, spleen, pancreas, kidney, testicle were normal. Chest enhanced CT scan revealed mild inflammation in left lower lobe.

For treatment of AE with coexistent anti-LGI1 and anti-GABA_B_R1, he received 1 g and 0.5 g intravenous methylprednisolone separately, 3 days for each dosage, and then remained on an oral steroid taper for half year. After intravenous and oral sodium supplement, blood sodium and chlorine gradually increased to normal (Table 1). His symptoms improved greatly and EEG recovered to normal.

**Table 1:** Blood test for sodium and chlorine

Date/time	Blood sodium(mmol/L)	Blood chlorine(mmol/L)
2020-04-21 11:00	119.0	81.0
2020-04-21 20:00	121.9	82.0
2020-04-22	124.0	85.0
2020-04-23	129.0	92.0
2020-04-24	135.0	93.0
2020-04-26	138.0	92.0
2020-04-29	134.0	91.0
2020-04-30	135.0	91.0

## Discussion and conclusion

Here we report the first case of AE with coexistent serum anti-LGI1 and anti- GABA_B_R1. The 60-year-old male, with subacute onset, mainly manifested cognitive decline, behavioral abnormality, insomnia, refractory hyponatremia, abnormal EEG, and positive anti-LGI1 and anti-GABA_B_R1 in serum. According to research and clinical guidelines [6], the cognitive decline, behavioral abnormality and seizures of the patient are the common clinical manifestations of LGI1-AE and GABABR1-AE, but sleep disorder is more common in LGI1-AE, and hyponatremia is unique to LGI1-AE. So, the clinical characteristics of this case were more inclined to anti-LGI1 encephalitis. The patient responded well to glucocorticoid treatment, and we will continue to follow up the prognosis.

LGI1 is a secretory protein mainly expressed in the hippocampus and neocortex, that connects presynaptic epilepsy-related ADAM23 to postsynaptic ADAM22. Anti-LGI1 interrupts the inhibitory signal transmission from the presynaptic potassium channel to the postsynaptic AMPA receptors, thus increasing the excitability of nerve tissue and resulting epilepsy or encephalitis. LGI1-AE, mostly found in elderly men with subacute onset, can demonstrate cognitive impairment, behavioral change, personality abnormality, hyponatremia and frequent seizures, characterized by facio-brachial dystonic seizures, while the majority of patients does not present with generalized tonic-clonic seizures. Most patients have no related tumors, only about 10% had thymoma, while other tumors were rare. Up to 75% of cases have normal CSF routine analysis. EEG can show mild diffuse slow wave, and about half may have swelled medial temporal lobe with high T2/flair signal. The good news is the relatively low recurrence rate [7, 8]. GABA_B_R regulates voltage-sensitive calcium channel and inward compensatory potassium channels through G protein. GABA_B_R is widely spread in brain and spine and particularly abundant in the hippocampus, thalamus and cerebellum. Anti-GABA_B_R is related to seizures, memory loss, anxiety and mood disorder. GABA_B_R1-AE mostly presents limbic encephalitis symptoms, with temporal lobe epilepsy as the core symptomatology, and most of them are accompanied by cognitive function decline, personality change and mental behavior abnormality. About 50% of patients have small cell lung cancer or neuroendocrine tumor. It is suggested that anti-GABA_B_R encephalitis should further take chest CT or PET examination [9].

The overlying of neuronal auto-antibodies may cause the superposition of clinical syndrome, but not a simple complete superposition, which needs to be analyzed according to the specific antibody type and clinical manifestation. According to Professor Guan Hongzhi’s newly published review, it is necessary to distinguish whether the antibodies in patients belong to pathogenic markers or concomitant antibodies [10]. The main manifestations of this case are psychobehavioral abnormality and hyponatremia, more similar to clinical manifestations of anti-LGI1 AE.

The co-existence of multiple auto-antibody is rare (summarized in Table 2). Ren Haitao reported 531 cases of AE with auto-antibodies, and only 10 cases detected multiple anti-neuronal antibodies, among whom 5 cases were anti-GABA_B_R/anti-Hu, 1 anti-NMDAR/APQ-4, 1 anti-LGI1/anti-CASPR2, 1 anti-LGI1/anti-Yo, 1 anti-AMPAR/anti-CV2 and 1 anti-AMPAR/anti-Hu [10]. In the 20 anti-GABA_B_R-AE cases reported by Hoftberger, 7 detected multiple auto-antibodies, among whom 3 cases with anti-Sox1, 1 with anti-Ri, 1 with anti-amphiphysin, 1 with anti-GAD65 and 1 with anti-NMDAR [11]. Liu XY recently reported one case characterized by double positive of anti-LGI1 and anti-NMDAR [12]. Boronat reported a case of anti-GABA_B_R combined with anti-GAD65, manifesting cerebellar ataxia and thyroid carcinoid [13]. Qi Hengchang reported two cases of AE with multiple auto-antibodies against neuron (one was anti-NMDAR/anti-GABA_B_R, and the other anti-LGI1/anti CASPR2. Both patients were adult women with acute onset. Their first symptom was epilepsy, and the treatment effect was good [14]. Wang XJ retrospectively analyzed 255 AE patients from our hospital and found 7 cases with multiple autoantibodies [15]. Also, Qiu ZD reported 6 AE with coexistent autoantibodies out of 134 cases [16]. Chung recently described a patient with antibodies to GABAB and IgLON5, who presented with sleep disorders like our case [17].

**Table 2:** Clinical data of AE cases with multiple auto-antibodies

N.	Sex, age	AE auto-Abs		Other Abs		Clinical manifestations	Brain MRI	tumor	prognosis
		serum	CSF	serum	CSF				
1	M,62	GABA_B_R	GABA_B_R	Hu	–	Memory loss, somnolence, conculsion, cough, hoarseness	Normal	Lung cancer	Improve
2	M,61	GABA_B_R	GABA_B_R	Hu	–	Epilepsy, somnolence, memory loss	Normal	Lung cancer	Improve
3	M,59	GABA_B_R	GABA_B_R	Hu	Hu	Epilepsy, psychosis	Lesions of bilateral hippocampus	Lung cancer	Improve
4	M,58	GABA_B_R	GABA_B_R	Hu	Hu	Psychosis, memory loss, numbness of limbs	Lesions of bilateral hippocampus	Lung cancer	Improve
5	M,61	GABA_B_R	GABA_B_R NMDAR	Hu	–	Epilepsy, memory loss, coma	ND	Lung cancer	Improve
6	F,19	–	NMDAR	AQP4	AQP4	Psychosis, memory loss, blepharoptosis	Lesions of bilateral basal ganglia, brainstem	No	Improve
7	F,40	LGI1 CASPR2	LGI1	–	–	Myalgia, fasciculation, epilepsy, insomnia	Normal	No	Improve
8	F,56	LGI1	LGI1	Yo	Yo	Memory loss, conculsion, somnolence, polyphagia	Normal	No	Improve
9	F,50	AMPAR	AMPAR	CV2	CV2	Memory loss, psychosis	Normal	Thymoma	Improve
10	F,51	AMPAR	AMPAR	Hu	–	Psychosis, dysphagia, dysdipsia	Lesions of bilateral cortex	Mediastinal occupying	Dead
11	M,44	ND	GABA_B_R NMDAR	–	–	Limbic encephalitis	Not mentioned	No	Complete improve
12	F,63	–	GABA_B_R		GAD65	Status epilepticus	Not mentioned	No	Dead
13	M,60	GABA_B_R	GABA_B_R		SOX1^*^	Limbic encephalitis	Not mentioned	SCLC	Partial recovery
14	M,62	GABA_B_R	GABA_B_R		Ri^*^	Limbic encephalitis	Not mentioned	SCLC	–
15	F,68	GABA_B_R	GABA_B_R		SOX1^*^	Limbic encephalitis	Not mentioned	SCLC	Partial recovery
16	M,74	GABA_B_R	ND	SOX1	ND	Limbic encephalitis	Not mentioned	SCLC	Dead
17	M,77	GABA_B_R	GABA_B_R		Amphiphysin^*^	Limbic encephalitis	Not mentioned	SCLC	Unresponsive
18	F,57	LGI1 NMDAR	–	–	–	Faciobrachial dystonic seizure, hyponatremia, mental disorder	–	No	Improve
19	M,66	GABA_B_R^*^		GAD^*^		Seizures, confusion	Normal	SCLC	Not available
20	M,47	GABA_B_R^*^		SOX1^*^VGKC		Seizures, behavior change, memory impairment	Bilateral temporal lesions	SCLC	Partial recovery
21	M,70	GABA_B_R^*^		GAD^*^ SOX1		Seizures, memory impairment, confusion	Normal	SCLC	Unresponsive, dead
22	M,58	GABA_B_R^*^		Hu^*^		Seizures, memory impairment	Bilateral temporal lesions	SCLC	Unresponsive, dead
23	M,61	GABA_B_R^*^		BRSK2^*^		Memory impairment	Bilateral temporal lesions	SCLC	Unresponsive
24	F,57	GABA_B_R^*^		GAD^*^		Subacute cerebellar ataxia	Normal	Carcinoid of thymus	Complete recovery
25	F,30	NMDAR GABA_B_R	NMDAR	–	–	Epilepsy, psychosis, insomnia	Normal	No	Improve
26	F,43	LGI1 CASPR2	CASPR2	–	–	Seizures, weight loss, calculation/ memory/speech disorder	Bilateral hippocampus/ occipital/parietal lesions	No	Improve
27	F,67	LGI1		Hu		Memory loss, motor aphasia	Left frontal/temporal/ parietal/occipital lobe	No	Improve
28	F,57	NMDAR LGI1				Seizures, facio-brachial dystonic seizures, somnolence	Demyelination of white matter	Probable lung cancer, thyroid nodule 4th	Improve
29	F,84		GABA_B_R	Hu		Seizures, cognitive impairment, confusion, psychosis	Left hippocampus and temporal lobe	Probable lung cancer	Dead
30	M,55		NMDAR	Ma2		Sensory aphasia, memory loss	Left temporal/occipital lobe and hippocampus	No	Improve
31	F,60		GABA_B_R	amphiphsin		Seizures, cognitive impairment, memory loss	Bilateral hippocampus, right temporal lobe	SCLC	Dead
32	M,67	LGI1	LGI1 NMDAR			Seizures, phychosis, memory loss, hand groping, hyponatremia	Unable to cooperate	No	Imptove
33	F,43		NMDAR	Yo		Phychosis, seizures, memory loss	Bilateral hippocampus and temporal lobe	Myoma of uterus	Improve
34	F,82	GABA_B_R	GABA_B_R	amphiphsin	amphiphsin	Memory loss, psychosis, disorientation	Left temporal lobe and hippocampus	Breast cancer	Unchanged
35	F,62			SOX1 Titin	SOX1	Polyneuropathy, subacute cerebellar degeneration	–	Cholangiocarcinoma	Dead
36	M,72	GABA_B_R	GABA_B_R	Amphiphsin,Hu,GAD65	Amphiphsin,Hu,GAD65	Subacute sensory neuropathy, seizures, psychosis	–	Probable gastric cancer	Dead
37	M,62	LGI1	LGI1	Yo	Yo	Memory loss, seizures, dizziness	Bilateral hippocampus	–	Improve
38	M,70	LGI1 CASPR2				Weekness of limbs, cognitive impairment, barylalia	–	Probable carcinoid of thymus	Improve
39	M.62			Hu, Ri	Hu, Ri	Polyneuropathy	–	SCLC	Dead
40	M,58	IgLON5 GABA_B_R	IgLON5			Dysarthria, gait instability, apraxia, hallucination,sleep disorder	Normal	No	Improve

*Only serum was tested for auto-antibodies, and CSF was not detected

The clinical significance of multiple auto-antibody has already raised attention of many neurologists and needs to be interpreted in combination with clinical practice. For example, anti-GABA_B_R can be combined with anti-Hu. When anti-GABA_B_R is positive, it is recommended to screen anti-Hu and carry out tumor screening at the same time, such as chest CT, tumor markers, etc., excluding the possibility of tumor as much as possible. In this case chest enhanced CT scan didn’t find tumor, but the patient was advised to take regular examination during follow-up. However, since it’s a single case report, it might be a coincidence despite its great significance.

Here we first report an AE case with co-existing anti-LGI1 and anti-GABA_B_R1. The existence of concomitant autoantibodies should be considered when the patients exhibit atypical and overlapping symptoms. Caution should be given because it’s a single-case report. With the discovery of more multiple auto-antibody positive cases of AE, it will provide evidence for further revealing the clinical characteristics, treatment and prognosis.

## Data Availability

There are no associated datasets for this manuscript. All data generated or analyzed during this study are included in this published article. Related queries can be directed to the corresponding author.

## Abbreviations

LGI1: Leucinie-rich Glioma Inactivated 1
GABA_B_R: γ-aminobutyric acid-beta-receptor
NMDAR: N-methyl-D-aspartic acid receptor
AMPAR: α-amino-3-hydroxy-5-methyl-4-isoxazolepropionic acid receptor
CSF: Cerebrospinal fluid
AE: Autoimmune encephalitis
